# Integrated analysis of the association between methionine cycle and risk of moyamoya disease

**DOI:** 10.1111/cns.14254

**Published:** 2023-05-14

**Authors:** Junsheng Li, Qiheng He, Chenglong Liu, Chaofan Zeng, Chuming Tao, Yuanren Zhai, Wei Liu, Qian Zhang, Rong Wang, Yan Zhang, Peicong Ge, Dong Zhang, Jizong Zhao

**Affiliations:** ^1^ Department of Neurosurgery, Beijing Tiantan Hospital Capital Medical University Beijing China; ^2^ China National Clinical Research Center for Neurological Diseases Beijing China; ^3^ Department of Neurosurgery The Second Affiliated Hospital of Soochow University Suzhou China

**Keywords:** biomarker, metabolite, methionine cycle, Moyamoya disease, risk model

## Abstract

**Objective:**

The role of methionine (Met) cycle in the pathogenesis and progression of cardiovascular and cerebrovascular diseases has been established, but its association with moyamoya disease (MMD) has rarely been studied. This study aimed to analyze the levels of Met cycle‐related metabolites and constructed a risk model to explore its association with the risk of MMD.

**Methods:**

In this prospective study, a total of 302 adult MMD patients and 88 age‐matched healthy individuals were consecutively recruited. The serum levels of Met cycle‐related metabolites were quantified by liquid chromatography‐mass spectrometry (LC–MS). Participants were randomly divided into training set and testing set at a ratio of 1:1. The training set was used to construct the risk score model by LASSO regression. The association between Met cycle‐related risk score and the risk of MMD was analyzed using logistic regression and assessed by ROC curves. The testing set was used for validation.

**Results:**

The levels of methionine sulfoxide and homocysteine were significantly increased, while the levels of betaine and choline were significantly decreased in MMD and its subtypes compared to healthy controls (*p* < 0.05 for all). The training set was used to construct the risk model and the risk score of each participant has been calculated. After adjusting for potential confounders, the risk score was independently associated with the risk of MMD and its subtypes (*p* < 0.05 for all). We then divided the participants into low‐risk and high‐risk groups, the high‐risk score was significantly associated with the risk of MMD and its subtypes (*p* < 0.05 for all). The risk scores were further assessed as tertiles, the highest tertile was significantly associated with a higher risk of MMD and its subtypes compared to the lowest (*p* < 0.05 for all). The results were validated in the testing set.

**Conclusion:**

This study has constructed and validated a risk model based on Met cycle‐related metabolites, which was independently associated with the risk of MMD and its subtypes. The findings provided a new perspective on the risk evaluation and prevention of MMD.

## Graphical abstract

We constructed a risk score model based on Met cycle‐related metabolites to analyze the association between Met cycle dysregulation and the risk of MMD and validated it with a testing set.

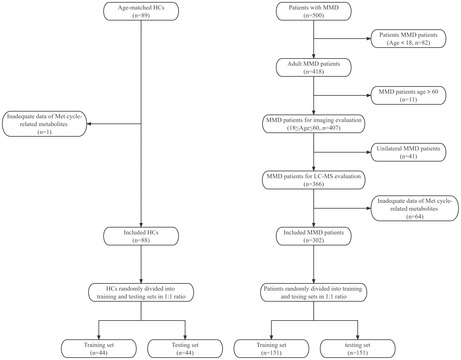

## 1 INTRODUCTION

Moyamoya disease (MMD) was a rare cerebrovascular disorder characterized by progressive stenosis or occlusion of the internal carotid arteries, leading to the formation of abnormal collateral vessels at the base of the brain.^1^ Patients with MMD commonly presented with a wide range of ischemic or hemorrhagic symptoms, including headache, seizure, cognitive decline, transient ischemic attack (TIA), infarction, and intracranial hemorrhage.^2^ Epidemiology showed that MMD was more prevalent in East Asian countries and exhibited a familial aggregation pattern, suggesting the genetic predisposition.^3^, ^4^ Although various risk factors have been identified, including RNF213 variants, immune response, and environmental factors, the underlying pathophysiology of MMD remained poorly understood.^5^, ^6^ Current therapeutic approaches for MMD focus on revascularization procedures using extracranial vessels to restore the blood supply of the affected hemispheres.^7^ However, the outcomes of surgical interventions and the long‐term prognosis of patients exhibit a considerable inter‐individual variability. Hence, there has been a critical need to identify the potential factors associated with the risk of MMD and develop targeted prevention strategies for at‐risk populations. Further studies are needed to improve the understanding of this complex disease and advance the development of more effective treatments.

Metabolomics provided a comprehensive understanding of the metabolic state of organisms.^8^ Analyses of the pattern and level of metabolites contributed to identifying the disease‐related changes, which provided a unique insight into the underlying biological processes.^9^, ^10^ In recent years, metabolomics has become an increasingly powerful tool in the identification and validation of biomarkers for various diseases. Methionine (Met) cycle was a complex metabolic pathway with important regulatory functions in cellular processes such as DNA synthesis, methylation, and redox balance.^11^, ^12^, ^13^ The altered levels of Met cycle‐related metabolites played a crucial role in the pathogenesis and progression of cardiovascular and cerebrovascular diseases.^14^, ^15^ The correlation between homocysteine (Hcy) and MMD has been discussed in previous studies.^16^, ^17^ Furthermore, the association between decreased levels of choline and betaine and the risk of MMD has been recently identified.^18^ It indicated the complex interactions between the Met cycle and the risk of MMD. However, the integrated analysis of the role of Met cycle‐related metabolites in MMD has hardly been studied.

Therefore, we analyzed the levels of Met cycle‐related metabolites in MMD and constructed a Met cycle‐related risk model, which helped to demonstrate the correlation between Met cycle and the risk of MMD. Our study suggested that interventions targeting the Met cycle might have therapeutic potential in the prevention and treatment of MMD.

## 2 METHODS

### 2.1 Participants and study design

**FIGURE 1:**
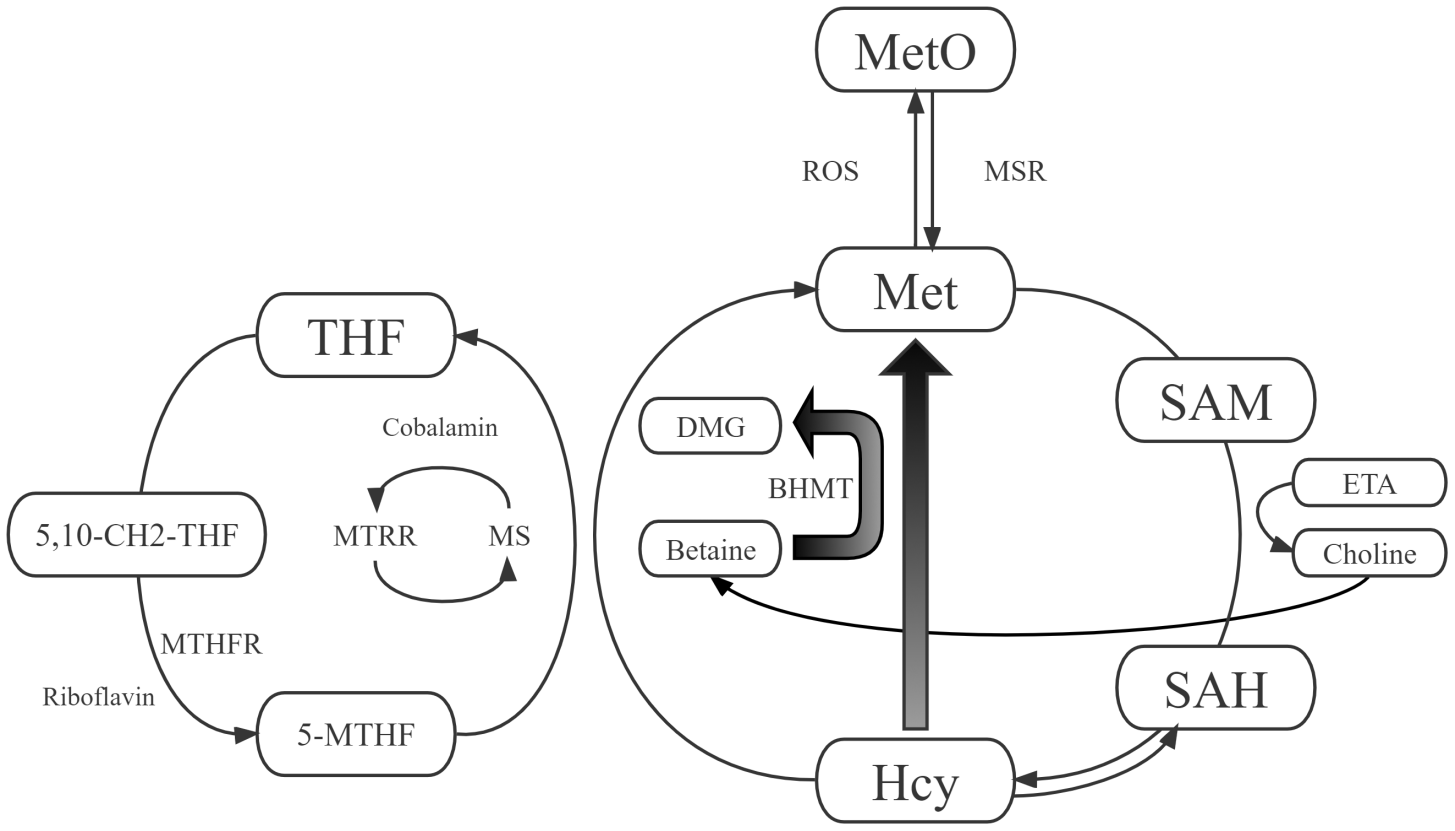
Key metabolites involved in methionine cycle.

**FIGURE 2:**
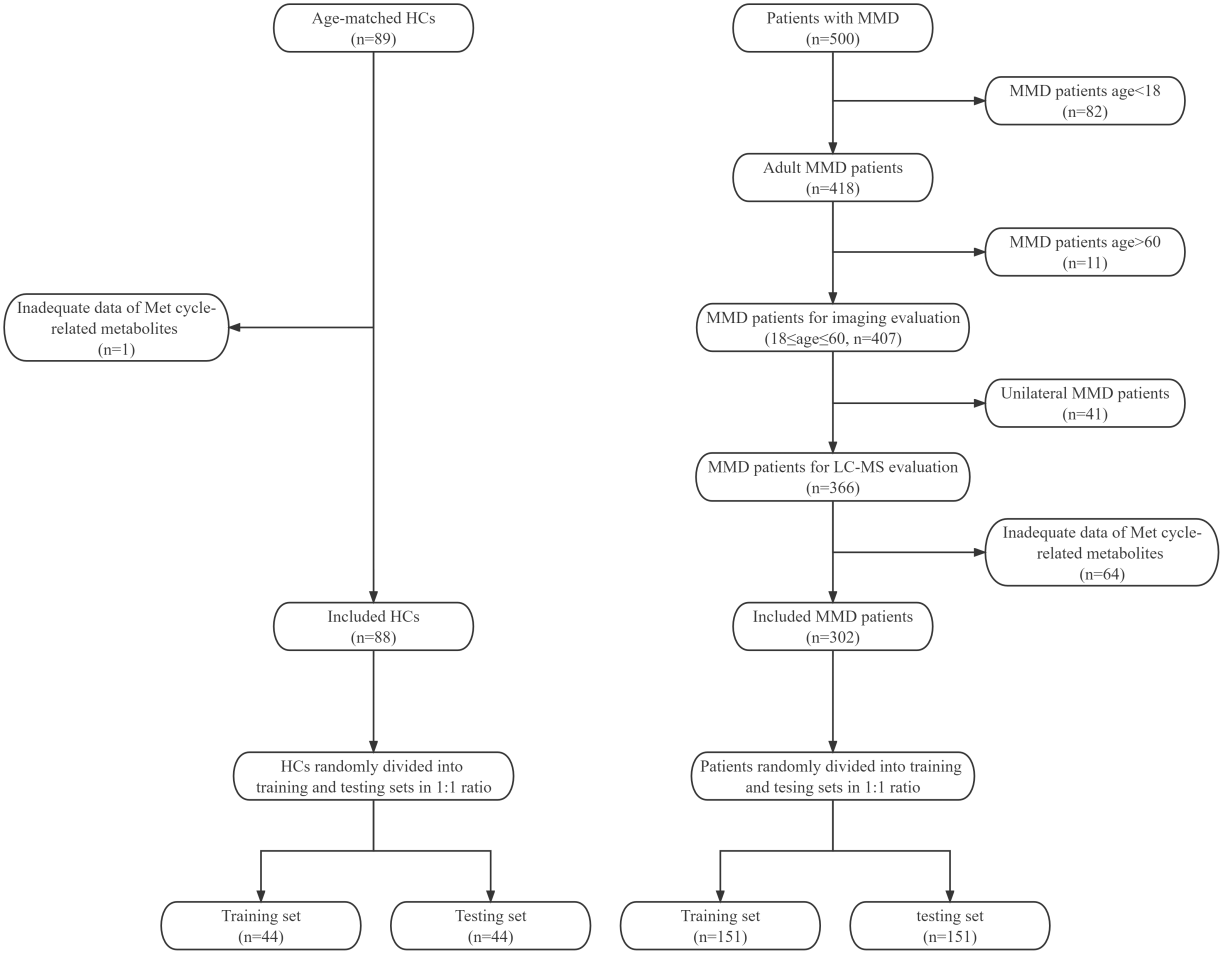
Flowchart of participant recruitment.

In this prospective study, we consecutively recruited patients diagnosed with MMD using digital subtraction angiography (DSA) according to the 2012 Japanese diagnostic criteria from September 2020 to December 2021 at Beijing Tiantan Hospital.^19^ Minors and unilateral MMD patients were excluded from analyses. We also included a control group of 89 age‐matched individuals without any underlying diseases or cerebrovascular diseases. After obtaining written informed consent, we collected the peripheral blood samples and information on demographics, clinical characteristics, and laboratory examination of the participants. Blood samples were tested using liquid chromatography‐mass spectrometry (LC–MS) to quantify levels of methionine cycle‐related metabolites (Figure 1). Participants without complete LC–MS data were excluded from the study. We then randomly divided all participants into training set and testing set at a ratio of 1:1. The participant recruitment process has been illustrated (Figure 2). The Ethics Committee of Beijing Tiantan Hospital has reviewed and approved this study (KY2022‐051‐02).

### 2.2 Data collection

We collected the baseline patient characteristics, including age, gender, past medical history, heart rate, systolic blood pressure (SBP), diastolic blood pressure (DBP), body mass index (BMI), RNF213 variants, and laboratory tests. Fasting peripheral venous blood samples were obtained with vacuum biochemical tubes on the second morning after admission. All samples were centrifuged, aliquoted, and stored at −80°C within 1 h after collection. In order to avoid bias in the test results, all information of participants was blinded to the laboratory technicians. RNF213 p.R4810K variants were confirmed with the following primer sequences: Forward 5′‐GCCCTCCATTTCTAGCACAC‐3′ and Reverse 5′‐AGCTGTGGCGAAAGCTTCTA‐3′. Neurological function evaluation at admission was assessed using the modified Rankin Scale (mRS) scores, which were divided into two levels (0–2 and 3–5). The severity of MMD patients was determined based on the Suzuki stage of the more severe side.

### 2.3 Construction of Met cycle‐related risk model

The levels of Met cycle‐related metabolites in the training set were incorporated into LASSO regression to construct the risk model. The lambda that minimized the deviations has been selected. Based on the levels of Met cycle‐related metabolites, a risk score was calculated for each participant using the following formula (*n*, metabolite quantity; level_*i*_, metabolite level; coefficient_*i*_, metabolite regression coefficient)

$$\text{Risk score}=\sum_{i=1}^{n}{\text{level}}_{i}\times {\text{coefficient}}_{i}$$

### 2.4 Statistical analysis

The SPSS software (version 26.0) and R project (version 3.6.3) were used for the statistical analyses in this study. Continuous variables were analyzed for normality of the distribution using the Kolmogorov–Smirnov test. Normally distributed continuous variables were compared by *t*‐test and analysis of variance (ANOVA). The Bonferroni post hoc test was used as a post hoc test following ANOVA. Unordered categorical variables were analyzed using Pearson chi‐square test and Fisher exact test. Mann–Whitney *U* test and Kruskal–Wallis test were applied for non‐normally distributed continuous variables and ordered categorical variables. Logistic regression models were used to identify the risk factors associated with MMD and its subtypes. Crude model analyzed the risk score only. In Model 1, age, gender, heart rate, SBP, DBP, and BMI were added to crude model. Then WBC count, LY count, monocyte count, neutrophil count, PLT count, GLU, ALB, Cr, UA, TG, TC, HDL, LDL, ApoA, ApoB, and eGFR were further included for Model 2.^18^, ^20^ The testing set was used for validation. Receiver‐operating characteristic (ROC) curves and the area under ROC curve (AUC) were used to assess the predictive abilities of models for the risk of MMD and its subtypes. A two‐sided *p* value less than 0.05 was considered significant.

## 3 RESULTS

### 3.1 Study participants and baseline information

A total of 390 individuals were eventually enrolled in this study, consisting of 88 HCs and 302 MMD patients. Baseline characteristics between the two groups were compared, revealing that MMD patients had a higher prevalence of hypertension, diabetes, hyperlipidemia, smoking, and drinking, as well as higher levels of SBP, DBP, and BMI than HCs (*p* < 0.05 for all, Table 1). Additionally, it showed significantly higher levels of WBC count, NEUT count, and TG and lower levels of TC, HDL‐C, LDL‐C, and ApoA in the MMD group compared to the HC group. Furthermore, levels of Met cycle‐related metabolites were also analyzed, and found that the levels of methionine sulfoxide (MetO) and Hcy were significantly increased in the MMD group, while betaine and choline levels were significantly decreased (*p* < 0.05 for all).

**TABLE 1:** Baseline characteristics between HC and MMD groups.

Variables	HC (*n* = 88)	MMD (*n* = 302)	*p* Value
Age, years, mean ± SD	39.75 ± 11.62	41.70 ± 10.35	0.159
Gender, female, *n* (%)	51 (58.0)	179 (59.3)	0.825
*Medical history*			
Hypertension, *n* (%)	0 (0)	111 (36.8)	<0.001*
Diabetes, *n* (%)	0 (0)	48 (15.9)	<0.001*
Hyperlipidemia, *n* (%)	0 (0)	45 (14.9)	<0.001*
Smoking, *n* (%)	2 (2.3)	58 (19.2)	<0.001*
Drinking, *n* (%)	0 (0)	33 (10.9)	0.001*
*Clinical features, mean ± SD*			
Heart rate, bpm	77.75 ± 9.78	78.22 ± 6.16	0.669
SBP, mmHg	123.48 ± 11.74	132.47 ± 12.71	<0.001*
DBP, mmHg	78.22 ± 8.07	81.72 ± 9.48	0.001*
BMI, kg/m^2^	23.99 ± 3.39	25.60 ± 4.65	0.003*
*RNF213 p.R4810K, n (%)*			<0.001*
Wild‐type	88 (100)	219 (79.9)	
Mutant	0 (0)	55 (20.1)	
*Laboratory examinations, median (IQR)*			
WBC count, 10^9^/L	6.03 (1.90)	6.81 (2.47)	<0.001*
LY count, 10^9^/L	1.91 (0.72)	1.93 (0.89)	0.140
NEUT count, 10^9^/L	3.43 (1.62)	4.18 (1.94)	<0.001*
MONO count, 10^9^/L	0.35 (0.14)	0.36 (0.17)	0.264
PLT count, 10^9^/L	233.50 (90.50)	248.00 (71.75)	0.240
GLU, mmol/L	5.04 (0.62)	5.11 (1.05)	0.170
ALB, g/L	44.95 (3.18)	45.40 (3.83)	0.465
Cr, μmol/L	57.80 (19.35)	54.35 (20.53)	0.124
UA, μmol/L	310.50 (103.93)	306.00 (118.88)	0.680
TG, mmol/L	0.86 (0.62)	1.20 (0.83)	<0.001*
TC, mmol/L	4.61 (0.95)	4.23 (1.26)	<0.001*
HDL‐C, mmol/L	1.53 (0.40)	1.31 (0.36)	<0.001*
LDL‐C, mmol/L	2.69 (0.85)	2.40 (1.13)	0.003*
ApoA, g/L	1.39 (0.27)	1.30 (0.30)	0.002*
ApoB, g/L	0.77 (0.26)	0.82 (0.27)	0.181
eGFR, mL/min/1.73 m^2^	124.61 (22.72)	123.61 (19.70)	0.947
*Methionine cycle‐related metabolites, median (IQR)*			
Met, μmol/L	3.41 (1.52)	3.56 (1.50)	0.063
MetO, μmol/L	2.40 (0.22)	2.51 (0.34)	<0.001*
SAH, μmol/L	0.62 (0.07)	0.62 (0.08)	0.596
Hcy, μmol/L	10.61 (3.98)	11.59 (5.85)	0.003*
Betaine, μmol/L	40.15 (14.83)	31.35 (16.10)	<0.001*
DMG, μmol/L	3.57 (1.10)	3.86 (1.12)	0.054
ETA, μmol/L	3.23 (2.21)	3.52 (2.70)	0.124
Choline, μmol/L	12.70 (3.15)	10.60 (4.67)	<0.001*

Abbreviations: ALB, albumin; ApoA, apolipoprotein A; ApoB, apolipoprotein B; BMI, body mass index; Cr, creatinine; DBP, diastolic blood pressure; DMG, dimethylglycine; eGFR, estimated glomerular filtration rate; ETA, ethanolamine; GLU, glucose; HC, healthy control; Hcy, homocysteine; HDL‐C, high‐density lipoprotein cholesterol; IQR, interquartile range; LDL‐C, low‐density lipoprotein cholesterol; LY, lymphocyte; Met, methionine; MetO, methionine sulfoxide; MMD, moyamoya disease; MONO, monocyte; NEUT, neutrophil; PLT, Platelet; SAH, S‐adenosine homocysteine; SBP, systolic blood pressure; SD, standard deviation; TC, total cholesterol; TG, triglyceride; UA, uric acid; WBC, white blood cell.

* *p* < 0.05, significant difference.

Baseline characteristics between HCs and MMD subtypes (ischemic‐type and hemorrhagic‐type) were also compared. Both subtypes showed higher rates of hypertension, hyperlipidemia, smoking, and drinking, as well as higher levels of SBP compared to HCs (*p* < 0.05 for all, Table 2). In the ischemic MMD group, there were higher levels of WBC count, LY count, NEUT count, GLU and TG, and lower levels of TC, HDL‐C, LDL‐C, and ApoA compared to HCs (*p* < 0.05). In the hemorrhagic MMD group, there were higher levels of NEUT count, TG, and ApoB, and lower levels of HDL‐C and ApoA compared to HCs (*p* < 0.05 for all). Consistent with MMD overall, both ischemic and hemorrhagic subtypes showed significantly increased MetO and Hcy levels, and decreased betaine and choline levels (*p* < 0.05 for all).

**TABLE 2:** Baseline characteristics between HCs and MMD subtypes.

Variables	Health control (*n* = 88)	Ischemic MMD (*n* = 224)	*p* Value	Hemorrhagic MMD (*n* = 78)	*p* Value
Age, years, mean ± SD	39.75 ± 11.62	41.58 ± 10.16	0.198	42.04 ± 10.96	0.195
Gender, female, *n* (%)	51 (58.0)	127 (56.7)	0.840	52 (66.7)	0.248
*Medical history*					
Hypertension, *n* (%)	0 (0)	90 (40.2)	<0.001*	21 (26.9)	<0.001*
Diabetes, *n* (%)	0 (0)	45 (20.1)	<0.001*	3 (3.8)	0.102
Hyperlipidemia, *n* (%)	0 (0)	36 (16.1)	<0.001*	9 (11.5)	0.001*
Smoking, *n* (%)	2 (2.3)	46 (20.5)	<0.001*	12 (15.4)	0.002*
Drinking, *n* (%)	0 (0)	28 (12.5)	0.001*	5 (6.4)	0.021*
*Clinical features, mean ± SD*					
Heart rate, bpm	77.75 ± 9.78	78.03 ± 6.36	0.803	78.77 ± 5.54	0.404
SBP, mmHg	123.48 ± 11.74	133.79 ± 12.54	<0.001*	128.67 ± 12.53	0.007*
DBP, mmHg	78.22 ± 8.07	82.34 ± 9.65	<0.001*	80.31 ± 8.85	0.113
BMI, kg/m2	23.99 ± 3.39	25.96 ± 4.70	<0.001*	24.56 ± 4.36	0.350
*RNF213 p.R4810K, n (%)*			<0.001*		<0.001*
Wild‐type	88 (100)	162 (80.2)		57 (79.2)	
Mutant	0 (0)	40 (19.8)		15 (20.8)	
*Laboratory examinations, median (IQR)*					
WBC count, 10^9^/L	6.03 (1.90)	6.96 (8.19)	<0.001*	6.43 (7.84)	0.057
LY count, 10^9^/L	1.91 (0.72)	2.04 (2.49)	0.013*	1.73 (2.24)	0.193
NEUT count, 10^9^/L	3.43 (1.62)	4.26 (5.26)	<0.001*	3.85 (5.01)	0.021*
MONO count, 10^9^/L	0.35 (0.14)	0.36 (0.46)	0.135	0.35 (0.44)	0.919
PLT count, 10^9^/L	233.50 (90.50)	249.00 (286.00)	0.215	247.00 (279.25)	0.510
GLU, mmol/L	5.04 (0.62)	5.17 (5.91)	0.014*	4.92 (5.19)	0.116
ALB, g/L	44.95 (3.18)	45.50 (47.48)	0.344	45.20 (47.00)	0.997
Cr, μmol/L	57.80 (19.35)	55.30 (66.23)	0.223	52.60 (67.40)	0.072
UA, μmol/L	310.50 (103.93)	309.50 (378.88)	0.357	289.35 (355.35)	0.372
TG, mmol/L	0.86 (0.62)	1.22 (1.64)	<0.001*	1.17 (1.73)	0.008*
TC, mmol/L	4.61 (0.95)	4.17 (4.78)	<0.001*	4.41 (5.02)	0.422
HDL‐C, mmol/L	1.53 (0.40)	1.28 (1.49)	<0.001*	1.35 (1.54)	0.003*
LDL‐C, mmol/L	2.69 (0.85)	2.28 (2.92)	<0.001*	2.62 (3.30)	0.886
ApoA, g/L	1.39 (0.27)	1.30 (1.46)	0.002*	1.30 (1.47)	0.018*
ApoB, g/L	0.77 (0.26)	0.82 (0.96)	0.452	0.84 (1.01)	0.022*
eGFR mL/min/1.73 m^2^	124.61 (22.72)	124.01 (135.56)	0.823	121.78 (135.48)	0.750
*Methionine cycle‐related metabolites, median (IQR)*					
Met, μmol/L	3.41 (1.52)	3.55 (4.39)	0.072	3.60 (4.35)	0.153
MetO, μmol/L	2.40 (0.22)	2.49 (2.68)	<0.001*	2.54 (2.77)	<0.001*
SAH, μmol/L	0.62 (0.07)	0.62 (0.67)	0.725	0.63 (0.68)	0.436
Hcy, μmol/L	10.61 (3.98)	11.47 (15.25)	0.010*	12.00 (14.92)	0.004*
Betaine, μmol/L	40.15 (14.83)	32.45 (40.30)	<0.001*	29.55 (39.90)	<0.001*
DMG, μmol/L	3.57 (1.10)	3.85 (4.40)	0.088	3.88 (4.41)	0.064
ETA, μmol/L	3.23 (2.21)	3.51 (5.09)	0.112	3.56 (4.78)	0.348
Choline, μmol/L	12.70 (3.15)	10.90 (13.08)	<0.001*	9.84 (12.13)	<0.001*

* *p* < 0.05, significant difference.

### 3.2 Construction of Met cycle‐related risk model

**FIGURE 3:**
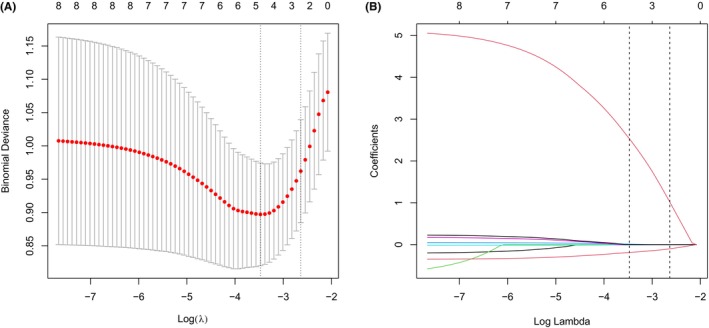
Construction of Met cycle‐related risk model. A. LASSO deviance profiles; B. LASSO coefficient profiles.

**FIGURE 4:**
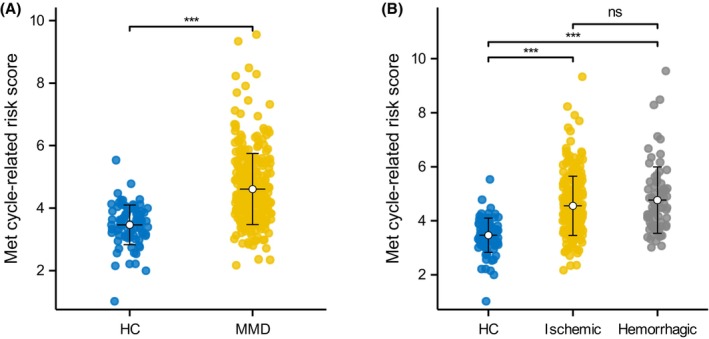
Levels of risk score between different groups. ns, not significant; ***p < 0.001. A. Risk score levels between MMD patients and HCs; B. Risk score levels between MMD subtypes and HCs.

All participants were randomly divided into training and testing sets, with equal numbers of 44 healthy individuals and 151 MMD patients in each set. The data of Met cycle‐related metabolites in training set was used to construct the risk score system by LASSO regression (Figure 3). Four metabolites were identified as the most significant contributors to the risk score, including MetO, Hcy, betaine, and choline (Table 3). The Met cycle‐related risk score was then calculated for all participants, and it was found to be significantly higher in MMD group and its subtype groups compared to the HC group (*p* < 0.001 for all, Figure 4).

**TABLE 3:** Coefficients in LASSO regression model of the Met cycle‐related metabolites.

Met cycle‐related metabolites	Odds ratio (95% CI)	*p* Value	Coefficients
Met	1.009 (0.755–1.350)	0.949	–
MetO	62.189 (8.017–482.378)	<0.001*	2.53356
SAH	0.818 (0.001–452.670)	0.950	–
Hcy	1.081 (1.004–1.164)	0.039*	0.01308
Betaine	0.963 (0.941–0.985)	0.001*	−0.00672
DMG	1.015 (0.781–1.319)	0.913	–
ETA	1.046 (0.865–1.264)	0.645	–
Choline	0.774 (0.682–0.878)	<0.001*	−0.18904

* *p* < 0.05, significant difference.

Based on the median risk score value, the MMD patients in training set were divided into low‐risk and high‐risk groups (Table 4). We compared the clinical characteristics of the two groups. It showed that there was a significantly higher proportion of hyperlipidemia in the high‐risk group (*p* = 0.013). The levels of WBC count (*p* = 0.026) and MONO count (*p* = 0.033) were also significantly higher in the high‐risk group than in the low‐risk group. Further division of MMD patients into low‐risk, intermediate‐risk, and high‐risk groups based on the tertiles of the risk score showed a significantly positive correlation between the risk score and hyperlipidemia (*r* = 0.204, *p* = 0.012, Table 5), levels of WBC count (*r* = 0.172, *p* = 0.035), and MONO count (*r* = 0.176, *p* = 0.031). Conversely, the level of HDL‐C was significantly negatively correlated with the risk score (*r* = −0.184, *p* = 0.024).

**TABLE 4:** Baseline characteristics of MMD patients in low and high‐risk groups.

Variables	Low risk (*n* = 75)	High risk (*n* = 76)	*p* Value
Age, years, mean ± SD	42.27 ± 8.79	41.62 ± 11.42	0.696
Gender, female, *n* (%)	45 (60.0)	41 (53.9)	0.453
*Medical history*			
Hypertension, *n* (%)	26 (34.7)	31 (40.8)	0.438
Diabetes, *n* (%)	13 (17.3)	12 (15.8)	0.799
Hyperlipidemia, *n* (%)	8 (10.7)	20 (26.3)	0.013*
Smoking, *n* (%)	17 (22.7)	13 (17.1)	0.392
Drinking, *n* (%)	10 (13.3)	8 (10.5)	0.595
*Clinical features, mean ± SD*			
Heart rate, bpm	76.79 ± 5.64	78.53 ± 5.95	0.067
SBP, mmHg	131.53 ± 10.94	133.62 ± 14.27	0.316
DBP, mmHg	81.33 ± 9.21	81.96 ± 10.57	0.698
BMI, kg/m^2^	25.11 ± 4.32	26.61 ± 5.36	0.060
*RNF213 p.R4810K, n (%)*			0.815
Wild‐type	56 (82.4)	59 (80.8)	
Mutant	12 (17.6)	14 (19.2)	
*Laboratory examinations, median (IQR)*			
WBC count, 10^9^/L	6.54 (2.27)	7.13 (2.18)	0.026*
LY count, 10^9^/L	1.89 (0.82)	2.14 (0.90)	0.189
NEUT count, 10^9^/L	3.78 (1.75)	4.25 (1.80)	0.078
MONO count, 10^9^/L	0.33 (0.14)	0.39 (0.17)	0.033*
PLT count, 10^9^/L	243.00 (74.00)	249.50 (86.25)	0.503
GLU, mmol/L	5.13 (1.09)	5.11 (1.14)	0.443
ALB, g/L	45.70 (4.50)	45.20 (3.25)	0.101
Cr, μmol/L	55.50 (20.50)	54.25 (22.35)	0.592
UA, μmol/L	293.80 (110.80)	321.10 (154.38)	0.361
TG, mmol/L	1.03 (0.80)	1.28 (0.74)	0.056
TC, mmol/L	4.26 (1.20)	4.20 (1.31)	0.554
HDL‐C, mmol/L	1.34 (0.38)	1.30 (0.34)	0.210
LDL‐C, mmol/L	2.40 (1.01)	2.37 (1.20)	0.935
ApoA, g/L	1.30 (0.31)	1.30 (0.38)	0.684
ApoB, g/L	0.84 (0.27)	0.85 (0.33)	0.673
eGFR, mL/min/1.73 m^2^	121.58 (18.39)	125.92 (24.09)	0.193
*Clinical characteristics, n (%)*			0.737
Ischemic‐type	69 (92.0)	71 (93.4)	
Hemorrhagic‐type	6 (8.0)	5 (6.6)	
*Admission mRS, n (%)*			0.593
0–2	59 (78.7)	57 (75.0)	
2–5	16 (21.3)	19 (25.0)	
*Suzuki stage, n (%)*			0.678
1–2	23 (30.7)	19 (25.0)	
3–4	38 (50.7)	44 (57.9)	
5–6	14 (18.7)	13 (17.1)	

*Note*: Suzuki's stage was defined on the more severe side.

* *p* < 0.05, significant difference.

**TABLE 5:** Baseline characteristics of MMD patients according to tertiles of the risk score.

Variables	Low risk (*n* = 50)	Intermediate risk (*n* = 50)	High risk (*n* = 51)	*p* Value	Spearman coefficient	*p* Value
Age, years, mean ± SD	42.64 (9.21)	41.50 (9.81)	41.69 (11.50)	0.836	−0.017	0.832
Gender, female, *n* (%)	30 (60.0)	31 (62.0)	25 (49.0)	0.365	0.091	0.265
*Medical history*						
Hypertension, *n* (%)	18 (36.0)	18 (36.0)	21 (41.2)	0.825	0.044	0.593
Diabetes, *n* (%)	8 (16.0)	9 (18.0)	8 (15.7)	0.944	−0.004	0.964
Hyperlipidemia, *n* (%)	4 (8.0)	10 (20.0)	14 (27.5)	0.040*	0.204	0.012*
Smoking, *n* (%)	10 (20.0)	8 (16.0)	12 (23.5)	0.638	0.037	0.654
Drinking, *n* (%)	5 (10.0)	7 (14.0)	6 (11.8)	0.826	0.022	0.790
*Clinical features, mean ± SD*						
Heart rate, bpm	77.50 (5.16)	77.34 (7.24)	78.14 (4.96)	0.771	0.068	0.405
SBP, mmHg	132.22 (12.02)	131.86 (14.51)	133.65 (11.68)	0.759	0.055	0.502
DBP, mmHg	81.12 (9.92)	82.08 (10.40)	81.75 (9.51)	0.887	0.057	0.486
BMI, kg/m^2^	24.58 (3.63)	26.52 (5.64)	26.50 (5.06)	0.074	0.136	0.097
*RNF213 p.R4810K, n (%)*				0.452	0.105	0.214
Wild‐type	38 (86.4)	38 (82.6)	39 (76.5)			
Mutant	6 (13.6)	8 (17.4)	12 (23.5)			
*Laboratory examinations, median (IQR)*						
WBC count, 10^9^/L	6.54 (2.23)	6.78 (2.42)	7.10 (2.03)	0.106	0.172	0.035*
LY count, 10^9^/L	1.91 (0.75)	1.87 (1.01)	2.15 (0.83)	0.267	0.126	0.124
NEUT count, 10^9^/L	3.87 (1.87)	3.74 (1.81)	4.25 (1.69)	0.206	0.145	0.075
MONO count, 10^9^/L	0.35 (0.15)	0.32 (0.25)	0.39 (0.13)	0.063	0.176	0.031*
PLT count, 10^9^/L	236.00 (74.00)	250.50 (79.00)	246.00 (89.00)	0.437	0.069	0.397
GLU, mmol/L	5.12 (0.86)	5.23 (1.71)	5.01 (0.60)	0.240	−0.118	0.149
ALB, g/L	45.85 (4.60)	45.30 (4.90)	45.20 (3.50)	0.338	−0.116	0.155
Cr, μmol/L	56.65 (20.40)	51.55 (21.73)	57.40 (23.20)	0.229	0.001	0.990
UA, μmol/L	293.45 (99.88)	294.90 (150.00)	330.10 (144.40)	0.132	0.144	0.077
TG, mmol/L	1.04 (0.84)	1.12 (0.78)	1.25 (0.71)	0.138	0.144	0.078
TC, mmol/L	4.47 (1.22)	4.19 (1.38)	4.16 (1.05)	0.221	−0.088	0.282
HDL‐C, mmol/L	1.34 (0.39)	1.32 (0.31)	1.27 (0.39)	0.077	−0.184	0.024*
LDL‐C, mmol/L	2.45 (1.09)	2.40 (1.03)	2.34 (1.09)	0.664	−0.015	0.856
ApoA, g/L	1.35 (0.32)	1.30 (0.35)	1.29 (0.35)	0.232	−0.133	0.104
ApoB, g/L	0.86 (0.28)	0.82 (0.30)	0.87 (0.31)	0.713	0.049	0.548
eGFR mL/min/1.73 m^2^	120.09 (19.22)	124.75 (20.18)	125.45 (25.40)	0.201	0.106	0.197
*Clinical characteristics, n (%)*				0.494	0.053	0.519
Ischemic‐type	41 (82.0)	36 (72.0)	39 (76.5)			
Hemorrhagic‐type	9 (18.0)	14 (28.0)	12 (23.5)			
*Admission mRS, n (%)*				0.482	−0.033	0.686
0–2	45 (90.0)	48 (96.0)	47 (92.2)			
2–5	5 (10.0)	2 (4.0)	4 (7.8)			
*Suzuki stage, n (%)*				0.420	−0.007	0.933
1–2	17 (34.0)	10 (20.0)	15 (29.4)			
3–4	23 (46.0)	30 (60.0)	29 (56.9)			
5–6	10 (20.0)	10 (20.0)	7 (13.7)			

*Note*: Suzuki's stage was defined on the more severe side.

* *p* < 0.05, significant difference.

### 3.3 Role of the risk score in MMD and its subtypes

**FIGURE 5:**
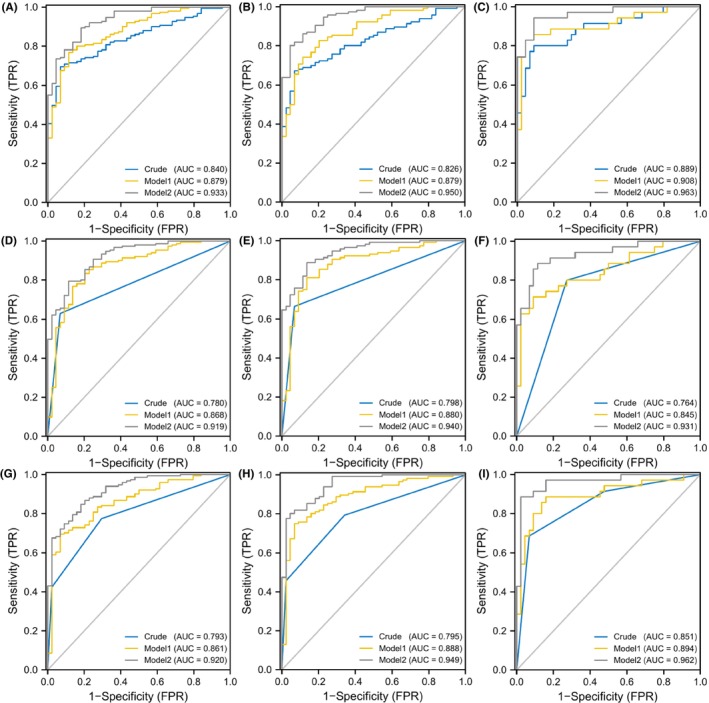
ROC curves of Met cycle‐related risk score in different models for the risk of MMD and its subtypes in training set. (A–C) ROC curves of Met cycle‐related risk score in different models for the risk of MMD and its subtypes. A. MMD overall; B. Ischemic‐type; C. Hemorrhagic‐type. (D–F) ROC curves of low and high levels of Met cycle‐related risk score in different models for the risk of MMD and its subtypes. D. MMD overall; E. Ischemic‐type; F. Hemorrhagic‐type. (G–I) ROC curves of Met cycle‐related risk score tertiles in different models for the risk of MMD and its subtypes. G. MMD overall; H. Ischemic‐type; I. Hemorrhagic‐type.

In the training set, logistic regression analyses showed a significant positive association between the Met cycle‐related risk score and the risk of MMD in Crude model (OR = 5.904, 95%CI = 3.153–11.057, *p* < 0.001, Table 6). After adjusting potential confounders, it showed that the risk of MMD enhanced with the increase in the risk score level in Model 1 (OR = 7.253, 95%CI = 3.380–15.566, *p* < 0.001) and Model 2 (OR = 11.322, 95%CI = 3.883–33.014, *p* < 0.001). The ROC curves indicated that Model 2 (AUC = 0.933, Figure 5A) prominently improved the predictive accuracy compared to the Crude model (AUC = 0.840) and Model 1 (AUC = 0.879). In addition, the risk score was also found to be independently associated with ischemic and hemorrhagic subtypes in all three models (Table 6). The ROC curves confirmed the predictive ability of these models (Figure 5B,C).

**TABLE 6:** Association between Met cycle‐related risk score and risk of MMD and its subtypes in the training set.

Met cycle‐related risk score	Crude model		Model 1		Model 2	
	OR (95% CI)	*p* Value	OR (95% CI)	*p* Value	OR (95% CI)	*p* Value
*MMD overall*						
Continuous	5.904 (3.153–11.057)	<0.001*	7.253 (3.380–15.566)	<0.001*	11.322 (3.883–33.014)	<0.001*
*Dichotomous*						
Low	Ref		Ref		Ref	
High	23.185 (6.859–78.363)	<0.001*	21.602 (6.098–76.526)	<0.001*	22.010 (5.285–91.655)	<0.001*
*Trichotomous*						
Low	Ref		Ref		Ref	
Intermediate	4.027 (1.821–8.903)	0.001*	5.018 (2.029–12.413)	<0.001*	5.745 (1.601–20.615)	0.007*
High	58.353 (7.631–446.220)	<0.001*	54.539 (6.851–434.177)	<0.001*	90.023 (8.306–975.730)	<0.001*
*Ischemic MMD*						
Continuous	5.328 (2.815–10.086)	<0.001*	6.634 (2.981–14.767)	<0.001*	12.214 (3.485–42.805)	<0.001*
*Dichotomous*						
Low	Ref		Ref		Ref	
High	26.983 (7.856–92.678)	<0.001*	24.531 (6.725–89.478)	<0.001*	23.769 (4.915–114.937)	<0.001*
*Trichotomous*						
Low	Ref		Ref		Ref	
Intermediate	3.366 (1.489–7.610)	0.004*	5.329 (1.875–15.145)	0.002*	12.832 (2.327–70.757)	0.003*
High	64.042 (8.236–497.950)	<0.001*	101.471 (10.716–960.817)	<0.001*	251.220 (13.695–4608.234)	<0.001*
*Hemorrhagic MMD*						
Continuous	15.763 (4.562–54.465)	<0.001*	23.775 (5.074–111.412)	<0.001*	100.369 (5.968–1688.136)	0.001*
*Dichotomous*						
Low	Ref		Ref		Ref	
High	10.667 (3.691–30.825)	<0.001*	13.555 (3.867–47.523)	<0.001*	92.783 (7.668–1122.659)	<0.001*
*Trichotomous*						
Low	Ref		Ref		Ref	
Intermediate	3.407 (0.789–14.720)	0.101	4.328 (0.829–22.601)	0.082	11.719 (0.741–185.382)	0.081
High	61.333 (11.211–335.543)	<0.001*	118.555 (14.078–998.379)	<0.001*	2154.885 (32.990–140754.809)	<0.001*

* *p* < 0.05, significant difference.

We then compared the risk of MMD between individuals with low and high‐risk scores and found that the high risk score was significantly associated with the higher risk of MMD in Crude model (OR = 23.185, 95%CI = 6.859–78.363, *p* < 0.001), Model 1 (OR = 21.602, 95%CI = 6.098–76.526, *p* < 0.001), and Model 2 (OR = 22.010, 95%CI = 5.285–91.655, *p* < 0.001). The predictive abilities of the models improved, as seen in the Crude model (AUC = 0.780, Figure 5D), Model 1 (AUC = 0.868), and Model 2 (AUC = 0.919). It showed similar results in Crude model, Model 1, and Model 2 of ischemic MMD and hemorrhagic MMD (Figure 5E,F).

We further classified the risk score into three levels based on the tertiles and analyzed the correlation between risk levels and MMD. As the risk level enhanced, the proportion of MMD events significantly increased (Table 6). Compared with the low‐risk individuals, those with intermediate‐risk and high‐risk had a significantly higher risk of MMD in all models (Crude model, intermediate‐risk: OR = 4.027, 95%CI = 1.821–8.903, *p* = 0.001, high‐risk: OR = 58.353, 95%CI = 7.631–446.220, *p* < 0.001; Model 1, intermediate‐risk: OR = 5.018, 95%CI = 2.029–12.413, *p* < 0.001, high‐risk: OR = 54.539, 95%CI = 6.851–434.177, *p* < 0.001; Model 2, intermediate‐risk: OR = 5.745, 95%CI = 1.601–20.615, *p* = 0.007, high‐risk: OR = 90.023, 95%CI = 8.306–975.730, *p* < 0.001). The AUC values increased with the models (Crude model, AUC = 0.793; Model 1, AUC = 0.861; Model 2, AUC = 0.920, Figure 5G). Consistent with MMD overall, the risk of ischemic MMD and hemorrhagic MMD significantly increased with the enhancement of risk levels. Although the effect was not statistically significant, an increased risk trend of hemorrhagic MMD was observed in the intermediate‐risk level compared with the low‐risk level. The AUC values of the ROC curves have been shown (Figure 5H,I).

**FIGURE 6:**
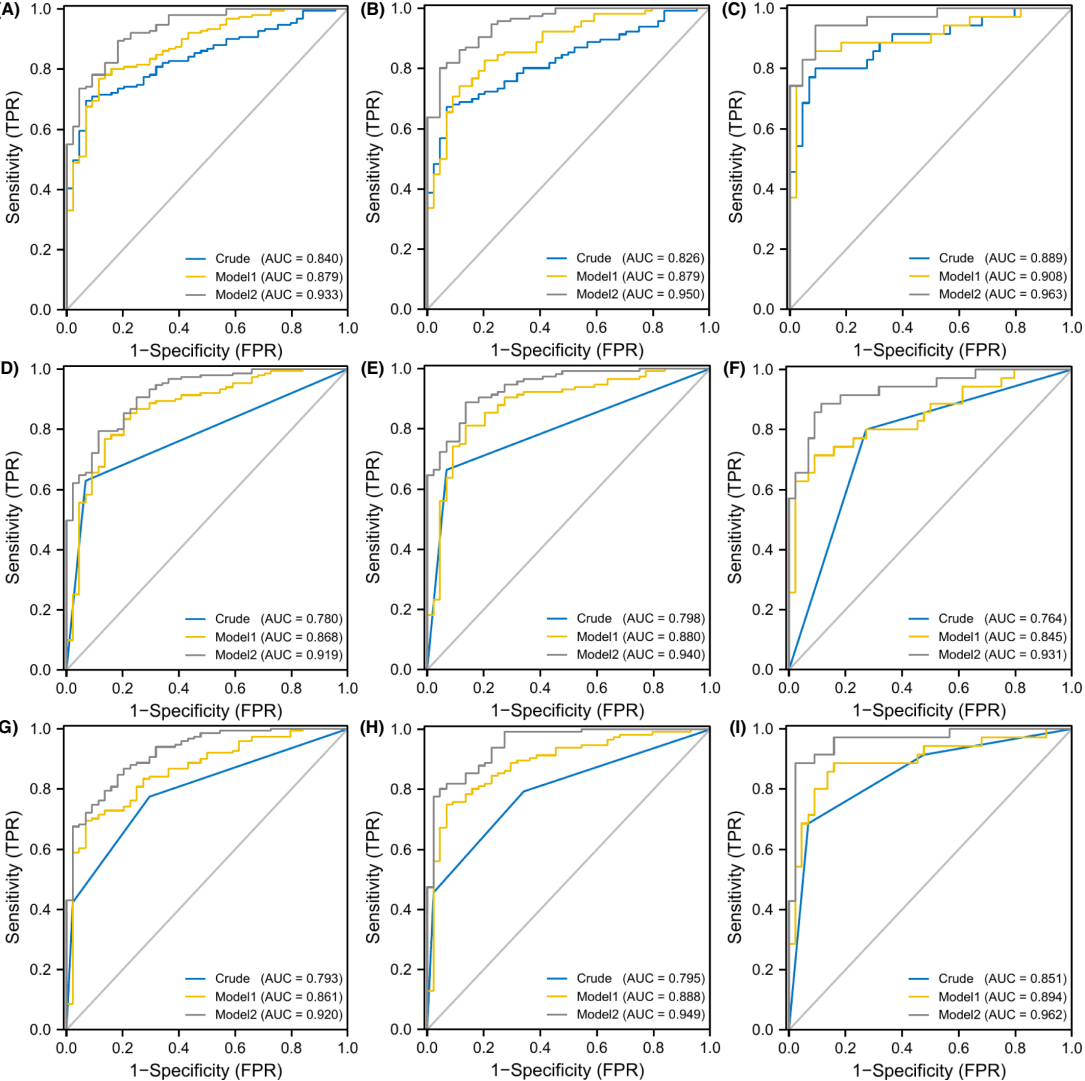
ROC curves of Met cycle‐related risk score in different models for the risk of MMD and its subtypes in testing set. (A–C) ROC curves of Met cycle‐related risk score in different models for the risk of MMD and its subtypes. A. MMD overall; B. Ischemic‐type; C. Hemorrhagic‐type. (D–F) ROC curves of low and high levels of Met cycle‐related risk score in different models for the risk of MMD and its subtypes. D. MMD overall; E. Ischemic‐type; F. Hemorrhagic‐type. (G–I) ROC curves of Met cycle‐related risk score tertiles in different models for the risk of MMD and its subtypes. G. MMD overall; H. Ischemic‐type; I. Hemorrhagic‐type.

We calculated the Met cycle‐related risk score of the individuals in the testing set and verified the association between the risk score and MMD. Subsequently, the participants in the testing set were then stratified into different risk levels based on the median value and tertiles of the risk score. The results confirmed that the risk score was independently associated with MMD and its subtypes (Table 7). The AUC values of the ROC curves confirmed the predictive accuracy of the models (Figure 6A–I).

**TABLE 7:** Association between Met cycle‐related risk score and risk of MMD and its subtypes in the testing set.

Met cycle‐related risk score	Crude model		Model 1		Model 2	
	OR (95% CI)	*p* Value	OR (95% CI)	*p* Value	OR (95% CI)	*p* Value
*MMD overall*						
Continuous	5.340 (2.781–10.255)	<0.001*	7.184 (3.268–15.791)	<0.001*	7.034 (2.693–18.374)	<0.001*
*Dichotomous*						
Low	Ref		Ref		Ref	
High	9.876 (3.932–24.805)	<0.001*	9.649 (3.700–25.166)	<0.001*	10.616 (3.241–34.773)	<0.001*
*Trichotomous*						
Low	Ref		Ref		Ref	
Intermediate	4.027 (1.821–8.903)	0.001*	4.839 (1.985–11.795)	0.001*	6.188 (1.877–20.398)	0.003*
High	58.353 (7.631‐446.220)	<0.001*	75.968 (9.210–626.608)	<0.001*	121.237 (10.775–1364.183)	<0.001*
*Ischemic MMD*						
Continuous	5.114 (2.594–10.082)	<0.001*	6.471 (2.853–14.676)	<0.001*	8.503 (2.638–27.407)	<0.001*
*Dichotomous*						
Low	Ref		Ref		Ref	
High	9.952 (3.872–25.578)	<0.001*	9.718 (3.489–27.069)	<0.001*	11.526 (2.958–44.919)	<0.001*
*Trichotomous*						
Low	Ref		Ref		Ref	
Intermediate	4.340 (1.892–9.955)	0.001*	5.513 (2.068–14.693)	0.001*	7.799 (1.593–38.191)	0.011*
High	50.840 (6.529‐395.906)	<0.001*	53.058 (6.328–444.881)	<0.001*	219.875 (12.090–3998.735)	<0.001*
*Hemorrhagic MMD*						
Continuous	6.622 (2.632–16.660)	<0.001*	7.472 (2.727–20.474)	<0.001*	32.847 (2.887–373.696)	0.005*
*Dichotomous*						
Low	Ref		Ref		Ref	
High	7.758 (2.989–20.132)	<0.001*	9.722 (3.364–28.096)	<0.001*	244.818 (8.595‐6973.352)	0.001*
*Trichotomous*						
Low	Ref		Ref		Ref	
Intermediate	3.115 (0.978–9.924)	0.055	4.204 (1.146–15.424)	0.030*	12.637 (1.123–142.176)	0.040*
High	18.400 (4.928–68.702)	<0.001*	22.989 (5.433–97.275)	<0.001*	323.372 (12.122‐8626.758)	0.001*

* *p* < 0.05, significant difference.

## 4 DISCUSSION

In this prospective study, we first compared the difference in clinical characteristics between MMD patients and HCs. We further explored the differences in ischemic and hemorrhagic MMD groups. It showed that the levels of four Met cycle‐related metabolites were significantly different in the MMD group and its subtypes from those in the HC group. The training set was used to construct the risk model by LASSO regression and analyze the role of Met cycle‐related risk score in MMD. We found that the risk score was independently associated with an increased risk of MMD and its subtypes. After adjusting for other risk factors, it showed an improvement in risk prediction. In this study, we provided a novel perspective on the role of metabolism dysregulation in the pathogenesis of MMD and suggested potential therapeutic targets.

One of the Met cycle‐related metabolites, MetO, was produced from Met by reactive oxygen species (ROS) and had the potential to serve as a biomarker of oxidative stress.^21^ MetO could be specifically reversed to Met by MetO reductase system.^22^ MetO has been shown to influence protein folding and stability, as well as protein–protein interactions, thereby impacting cellular functions such as oxidative stress, inflammation, and apoptosis. Growing evidence has suggested that the elevated level of MetO and dysfunction of MetO reductase system were associated with various diseases.^23^, ^24^, ^25^ Previous studies have discussed the relationship between MetO and cerebrovascular disease. A recent large‐scale prospective metabolomics study revealed that MetO was significantly associated with incident stroke, and its related metabolite score improved the prediction of the risk of incident ischemic stroke.^26^ Another study confirmed the protective role of MetO reductase system to neurovascular inflammation in ischemic stroke, which attenuated ROS‐augmented NF‐κB activation in endothelial cells and protected against the oxidation of Met residues in the regulatory domain of calcium/calmodulin‐dependent protein kinase II (CaMKII).^27^ Furthermore, the deficiency of MetO reductase A (MsrA) has been shown to promote vascular smooth muscle cell (VSMC) proliferation and neointimal hyperplasia through the extracellular signal‐regulated kinase 1/2 signaling pathway,^28^ which were also pathological changes associated with MMD. It suggested the role of MetO in the risk of MMD and MetO reductase system might be the potential therapeutic targets.^29^

Hcy, a sulfur‐containing amino acid, was a key determinant of Met cycle. Folate and vitamin B12 were involved in the remethylation of Hcy back into Met.^30^ Deficiency of these cofactors and mutation in methylenetetrahydrofolate reductase (MTHFR) and methionine synthase reductase (MTRR) could lead to excessive accumulation of plasma Hcy called hyperhomocysteinemia (HHcy).^31^ Previous studies found that Hcy was associated with cardiovascular diseases, ischemic stroke, neurological disorders, cognitive impairment, and cancer.^32^ In previous genome‐wide association study, researchers identified MTHFR and TCN2, two Hcy metabolism regulatory genes, as novel susceptibility genes for MMD, which were significantly associated with high‐serum Hcy levels in MMD.^33^ Previous case–control studies identified the positive association between Hcy, HHcy, and the risk of MMD.^16^, ^17^ Furthermore, HHcy has been found correlated to postoperative ischemia and poor postoperative angiogenesis in MMD patients.^34^, ^35^ Growing evidence has shown that MMD was associated with oxidative stress and chronic inflammation. Hcy could induce cellular and molecular oxidative injury via ROS and increase the level of MMP‐9 in the vascular wall.^36^, ^37^ In addition, the blocking effect of Hcy on nitric oxide (NO) synthesis has been shown to disrupt NO‐mediated endothelial‐dependent vasodilation, and the mitogenic effect of Hcy could lead to the proliferation of VSMCs.^38^ Hcy has also been linked to the promotion of coagulation and alterations in lipid metabolism, leading to thrombosis.^39^ These studies indicated the potential role of Hcy in the pathogenesis of MMD.

Choline has been an essential molecule involved in several key physiological processes, including the normal function of cell membranes, regulation of neurotransmitter function, transportation of lipids, and metabolism of one‐carbon units in body.^40^ Recent studies have shown a relationship between DNA methylation and the pathogenesis of MMD, with the aberrant promoter hypomethylation of Sortilin 1 as a novel biomarker.^41^, ^42^ We considered that the change in epigenetic patterns and regulation of gene expression by epigenetic modifications might be associated with the low intake of choline.^43^ Furthermore, plasma choline has been found inversely associated with cardiovascular events, recurrent stroke, and a lower risk of cognitive impairment after ischemic stroke.^44^, ^45^ Betaine, also known as trimethylglycine, was obtained directly from the diet or converted from choline by choline dehydrogenase and betaine aldehyde dehydrogenase. Betaine was an important methyl donor involved in the methylation process of Hcy into Met.^46^ An inadequate level of betaine could result in the accumulation of Hcy.^47^ Betaine played an important role in reducing oxidative stress, inhibiting NF‐κB activity, and NLRP3 inflammasome activation, and attenuating endoplasmic reticulum stress and apoptosis.^48^ Additionally, betaine has been shown to protect against coagulation events and reverse platelet aggregation.^49^ Interestingly, there seemed to be a potential link between betaine and MetO. A recent study has found that betaine could increase the expression of MetO reductases b1 (Msrb1) and b2 (Msrb2) to exert a neuroprotective effect in ischemia/reperfusion injury‐induced brain damage.^50^

The etiology of MMD remains unclear, which has been considered as a combined effect of immune, genetic, environmental, metabolic, and other factors. MMD has long been considered an irreversible condition with high disability and fatality rate, causing a huge burden to families and society. Direct and indirect revascularization procedures have been widely used in the treatment of MMD. However, the bypass surgery aimed to increase blood supply to the affected hemispheres, improve ischemic symptoms, and reduce the risk of recurrent hemorrhage, rather than reversing the progression of the disease. Therefore, it has been necessary to investigate novel risk factors associated with this disease, particularly modifiable risk factors, and explore potential therapeutic targets. Our study provided a new point of view in the exploration of metabolism dysregulation in the pathogenesis of MMD. In this study, we identified four different Met cycle‐related metabolites in MMD patients compared with HCs, including MetO, Hcy, choline, and betaine. We constructed a Met cycle‐related risk model with these metabolites and confirmed the correlation between the risk score and the risk of MMD. The Met cycle‐related risk score showed a favorable predictive ability in the risk of MMD and was validated by the testing set. However, there were still several limitations in our study. First, the sample size was relatively small due to the nature of a single‐center study. Therefore, the correlation between Met cycle dysregulation and the risk of MMD should be validated by large‐scale prospective studies. Second, the study only included adult patients with MMD, and it remained unclear whether the results could be extended to pediatric patients. Thirdly, dietary intake information was not collected, which might potentially impact the outcomes. Due to the limitation of the study design, we were unable to establish a causal link between Met cycle dysregulation and MMD, even after adjusting for potential confounding variables. Thus, further in vitro/in vivo experiments, as well as large‐scale prospective cohort studies with follow‐up outcomes, have been necessary to elucidate the effect and mechanism of Met cycle dysregulation on the pathogenesis of MMD.

## 5 CONCLUSION

In summary, our study has constructed and validated a risk model based on Met cycle‐related metabolites. We found that the Met cycle‐related risk score was independently associated with the risk of MMD and its subtypes. Our study provided a new perspective on the role of metabolism dysregulation in the pathogenesis of MMD and suggested potential therapeutic targets.

## AUTHOR CONTRIBUTIONS

Junsheng Li illustrated all the results and drafted the manuscript. Peicong Ge and Chaofan Zeng performed all the statistical analyses. Qiheng He, Chenglong Liu, Chuming Tao, and Yuanren Zhai collected the data. Wei Liu, Qian Zhang, Rong Wang, and Yan Zhang revised the manuscript. Dong Zhang and Jizong Zhao conceived and designed this research. All authors have read and approved the final version of the manuscript.

## FUNDING INFORMATION

This study was supported by the National Natural Science Foundation of China (81701137), the National Key Research and Development Program of China (2021YFC2500502), Beijing Municipal Organization Department Talents Project (2015000021469G219), and Beijing Municipal Administration of Hospitals' Mission Plan (SML20150501).

## CONFLICT OF INTEREST STATEMENT

The authors declared that no conflicts of interest existed.

## CONSENT TO PARTICIPATE

Written informed consent was obtained from all participants included in this study.

## Data Availability

All raw data supporting the findings in this study were available from the corresponding authors with reasonable requests.
